# Functional Characterization of Long Non-Coding RNAs Associated with Reproductive Fitness in Pura Raza Española Mares

**DOI:** 10.3390/ani16060898

**Published:** 2026-03-13

**Authors:** María Ángeles Vargas-Pérez, Nora Laseca, Sebastián Demyda-Peyrás, Mercedes Valera, Chiraz Ziadi, María Yuzhi Arjona-Delgado, Antonio Molina

**Affiliations:** 1 Departamento de Genética, Universidad de Córdoba, 14071 Córdoba, Spain; z72zizic@uco.es (C.Z.); b92ardem@uco.es (M.Y.A.-D.);; 2 Departamento de Agronomía, Escuela Técnica Superior de Ingeniería Agronómica, Universidad de Sevilla, Ctra. Utrera, Km 1, 41013 Sevilla, Spain; nlaseca@us.es (N.L.); mvalera@us.es (M.V.)

**Keywords:** long non-coding RNA, DNA:RNA triplex, fertility, reproductive efficiency

## Abstract

Long non-coding RNAs (lncRNAs) are transcripts constituted of more than 200 nucleotides that have been associated with the regulation of different biological processes by modulating the expression of key genes. In horses, evidence suggests that lncRNAs play a role in female reproductive fitness, yet their functional implications remain poorly characterized. The objective of this study was to investigate potential DNA:RNA triplex interactions between the promoter regions of fertility-related genes and lncRNAs transcribed from non-coding loci located within ±50 kb of these genes. By doing so, we aimed to elucidate the regulatory mechanisms underlying fertility in Pura Raza Española (PRE) horses. The observed distances (1.2–49.8 kb) were consistent with cis-acting lncRNAs. Furthermore, genomic context and structural accessibility analyses revealed that some predicted DNA-binding sites reside within CpG islands. This strategic localization in active promoter regions points toward a regulatory mechanism where lncRNAs may modulate transcriptional activity via triplex formation. Our results provide a concrete set of biologically plausible lncRNAs within fertility-associated genomic regions, representing targets for further functional validation and potential applications in genomic improvement strategies.

## Simple Summary

Reproductive fitness is a key factor in the success of equine breeding programs. However, the molecular mechanisms underlying fertility in mares remain largely unknown. In this study, we focus on long non-coding RNAs, non-coding transcripts that have been recognized as crucial regulators of gene expression. One mechanism through which long non-coding RNAs can regulate gene activity is the formation of DNA:RNA triplex structures. Using computational methods, we analyzed previously published genomic data to predict which long non-coding RNAs could bind to genes known to influence reproductive efficiency in Pura Raza Española mares via triplex formation. The analysis revealed several potential regulatory interactions, mostly found in regions of the genome known as CpG islands, which are often associated with gene promoters and play a key role in the epigenetic regulation of gene expression. These results suggest that long non-coding RNAs may modulate the expression of fertility-related genes through the recruitment of epigenetic regulators. Characterizing these molecular interactions could advance our understanding of the regulatory mechanisms underlying reproductive fitness in horses and identify non-coding regions that may contribute to functionally informed genomic prediction, subject to empirical validation.

## 1. Introduction

Reproduction is fundamental for the production, conservation, and genetic improvement of equines. In this context, reproductive aptitude in mares, including parameters such as age at first foaling, foaling interval, reproductive efficiency, and productive longevity, determines both the economic profitability of the studs and the demographic sustainability of the population. Genetic selection plays a key role in maintaining optimal fertility rates. It also minimizes reproductive dormancy and shortens the breeding cycle. However, the equine species exhibits unique physiological challenges, such as pronounced reproductive seasonality, low prolificacy, and a high energetic cost per offspring produced. These factors underscore the importance of maximizing reproductive efficiency in breeding programs [1].

Nevertheless, studying the genetic basis of reproductive aptitude in mares is constrained by multiple technical and biological limitations. First, reproductive traits typically show low to moderate heritability, which limits genetic progress through traditional selection [2,3]. Second, the reliability of genetic evaluations is compromised by the scarcity of complete phenotypic records and selection bias. It is also affected by the limited number of offspring per breeding female [4]. Third, the population structure of equine breeds is often closed and presents variable levels of inbreeding, making it difficult to disentangle genetic and environmental effects [2,3]. Therefore, traditional evaluations of genetic merit for reproductive traits tend to exhibit limited accuracy, hindering progress in this area [5,6].

Consequently, the identification of genomic markers for reproductive aptitude in mares emerges as a key strategy to promote breeding efficiency. The incorporation of genomic data into breeding programs increases the reliability of genetic evaluations while reducing generation intervals and dependence on phenotypic data [7]. In other species, genome-wide association studies (GWAS) have enabled the identification of regions associated with fertility, ovarian efficiency, or productive longevity [8,9]. However, genomic studies remain limited in horses, with only a few investigations addressing this topic. For instance, a GWAS in Pura Raza Española (PRE) mares identified multiple SNPs and candidate genes associated with reproductive efficiency [4]. Consequently, genomics in this species is still at an early stage, and so the characterization of functional markers beyond conventional SNPs would provide valuable insight into the underlying genetic mechanisms that determine reproductive aptitude.

From this perspective, long non-coding RNAs (lncRNAs) are highly promising candidates. lncRNAs are regulatory transcripts longer than 200 nucleotides that lack protein-coding capacity and regulate gene expression at the epigenetic, transcriptional, and post-transcriptional levels [10,11]. One mechanism involves the formation of DNA:RNA triplex structures with the major groove of the DNA double helix. This occurs through transient Hoogsteen interactions between nucleotide bases, providing an alternative, more flexible base pairing than the typical Watson–Crick hydrogen bonds. This binding is not random; it is concentrated at promoter regions, which are typically rich in CG dinucleotides, constituting CpG islands. Several studies have shown that lncRNAs interact with these regions to promote the recruitment of chromatin remodeling complexes that will further activate or repress the transcription of the target genes. For instance, the lncRNA *PARTICLE* forms a triplex with the CpG island of the *MAT2A* (S-adenosylmethionine synthase isoform type-2) promoter, recruiting the PRC2 (polycomb repressive complex 2) complexes to induce methylation and gene silencing. Another example is constituted by lncRNA *Khps1*, which acts as an anchor for a histone acetylase, mediating chromatin activation and thereby promoting *SPHK1* (Sphingosine kinase 1) gene expression. Thus, lncRNAs regulate gene expression of nearby or distant genes through both cis and trans mechanisms, respectively, by modulating the epigenetic status of CpG islands [10,12,13,14,15].

From a structural point of view, lncRNAs are usually characterized by a complex arrangement of loops comprising single-stranded and double-stranded sections that are organized into modules [16,17,18]. However, the DNA binding site in the lncRNA needs to be accessible to form triplexes. Ribonucleotides engaged in Watson–Crick interactions with each other are unavailable for the Hoogsteen interactions required with the double helix. Therefore, the integration of structural data into the triplex prediction pipeline has been proposed to be beneficial for improving accuracy [14,19,20].

Numerous studies in mammalian species have demonstrated that lncRNAs display tissue- and stage-specific expression patterns in reproductive organs, where they regulate key processes such as oocyte maturation, follicular development, corpus luteum function, and maternal-embryonic communication [21,22,23,24]. In horses, although studies remain scarce, a recent transcriptomic analysis has revealed differences in the uterine molecular landscape of fertile and subfertile mares [25]. These findings suggest that lncRNAs may play a relevant role in the regulation of fertility in this species.

In this context, the PRE breed represents an ideal model for the study of reproductive aptitude. With origins in Andalusia, the PRE is one of the most internationally recognized horse breeds, with more than 282,000 registered individuals and distribution across more than 70 countries [26]. Its breeding program, managed by the Asociación Nacional de Criadores de Caballos de Pura Raza Española (ANCCE), includes periodic genetic evaluations and a robust genealogical and phenotypic registry. Perdomo-González et al. [27] demonstrated the existence of parental effects in the transmission of reproductive aptitude, with both maternal and paternal gametic influences on traits such as age at first foaling, total number of foalings, and reproductive efficiency in PRE horses [27]. Similarly, Laseca et al. [3] reported that inbreeding depression negatively affects reproductive efficiency and longevity in PRE mares. These studies consolidate the breed as a reference model for investigating the genetic basis of equine reproduction.

A previous GWAS by Laseca et al. [4] identified several protein-coding genes as candidate modulators of reproductive efficiency in PRE mares. Building upon these findings, the present study aimed to explore the potential regulatory interactions between these specific target genes and lncRNAs, providing insights into the molecular mechanisms underlying reproductive fitness in PRE mares and identifying candidate functional markers that could be used in future genomic selection programs. To our knowledge, this is the first study to predict cis-acting DNA-lncRNA triplex interactions specifically associated with female fertility-related genes in the horse (*Equus caballus*).

## 2. Materials and Methods

### 2.1. Genome-Wide Association Study Analysis

This study integrated data from a previously published investigation on the PRE horse [4]. It employed a GWAS approach utilizing a univariate linear mixed model to identify genetic variants and candidate genes associated with mare fertility. Fertility was evaluated using the reproductive efficiency (RE) trait, which is defined as the percentual deviation between the optimal and real parity numbers of the mare at each age. RE encompassed all recorded foaling throughout the mare’s lifetime, up to her last known age, following the procedure described by Perdomo-Gonzalez et al. [6]. The GWAS was conducted on a robust cohort of 819 animals and controlled for population stratification using the first 10 principal components, achieving a genomic inflation factor of 0.999, which confirms the absence of statistical bias. Detailed information on this study is available in Laseca et al.’s work [4].

### 2.2. Definition of Genomic Windows and Identification of LncRNAs

The transcription start sites of target genes (TSS) were retrieved from the Ensembl database release 115 (genome assembly EquCab3.0, https://www.ensembl.org/index.html, accessed on 24 November 2025). A fixed genomic window spanning −50 kb to +50 kb relative to each TSS was defined for each gene. This threshold was selected based on Dhaka et al. [28], who demonstrated that windows larger than 50 kb are associated with a high false positive ratio. Furthermore, it is consistent with a recent transcriptomic analysis in Mongolian horses, where a ±50 kb window was used to identify potential target genes for differentially expressed lncRNAs [22]. lncRNA loci located in these windows were further selected using the biomaRt R package v2.64.0 [29,30], and their canonical transcript sequences were retrieved from the Ensembl database release 115.

To assess the potential functional impact of genetic variants, the genomic coordinates of the significant GWAS SNPs [4] were intersected with the loci of the identified lncRNAs using R package GenomicRanges v1.60.0 [31]. Co-localization was confirmed if the SNP position fell within the lncRNA locus regions.

Potential lncRNA-binding sites were defined as the regions extending from 3500 bp upstream to 1500 bp downstream of the TSS [32] to capture the core and distal mammalian promoter elements for each fertility candidate gene [4]. All genomic sequences were also downloaded from the Ensembl database release 115.

### 2.3. In Silico Prediction of Triplex Formation and LncRNA Accessibility

Triplexes established between lncRNAs and the upstream/downstream regions of the target genes were predicted by the command-line version of the software LongTarget v1.0 [33] with parameters -r 0 -c 5000 -i 60 -S 1.0 -ni 20 -na 100,000 -pc 0 -pt -1000 -ds 15 -lg 50, following the authors’ recommendations to prioritize high-affinity interactions while reducing the number of false-positive predictions generated by short random sequence matches. Moreover, permutation tests were applied using randomized target genes and lncRNA transcript sequences, as well as a negative control with the promoter region of the myostatin (*MSTN*) gene, all under the same parameter settings.

LongTarget considers all possible Hoogsteen base pairs that may be constituted between the lncRNA and the target DNA, together with their stabilities, identities and other biophysical measures, to identify the region with the highest density of predicted binding sites as the top candidate triplex-forming oligonucleotide (TFO), known as TFO1 [33]. Class 1 hits (i.e., top-ranking putative triplexes according to LongTarget) were further selected for downstream analysis to enhance the accuracy of triplex interaction prediction.

Putative lncRNA-binding regions in the DNA predicted by LongTarget were visualized on the Ensembl Genome Browser (Ensembl release 115) to identify overlaps with predicted CpG islands, which are regulatory regions involved in the epigenetic control of gene expression. CpG island coordinates from Ensembl release 115 and predicted lncRNA binding site positions from LongTarget output were converted into GRanges objects using R package GenomicRanges v1.60.0 [31]. Sites overlapping CpG islands were selected for downstream analysis.

Finally, RNAplfold 2.7.0 software [34] from the ViennaRNA package 2 (v2.7.0) [35] (https://github.com/ViennaRNA/ViennaRNA, accessed on 26 November 2025) was used to study the sequence accessibility of candidate lncRNAs. This accessibility is considered a prerequisite for triplex interactions with DNA, according to previous studies [19]. Default parameters were applied for the maximum base pair span and window size (−W 70 −L 70), consistent with previous observations that RNA molecules often fold locally during transcription, in a process known as co-transcriptional folding [36]. Therefore, local interactions are favored over long-range interactions. A threshold of 0.5 for unpaired probability (P_unp_) was applied to select highly exposed regions within the lncRNA sequences, as used by Matveishina et al. [19] to assess the structural accessibility of lncRNA *MEG3*. The P_unp_ value ranges from 0 to 1, where 0 indicates that the region is deeply buried within the secondary structure of the lncRNA, and 1 denotes total accessibility.

## 3. Results and Discussion

Exploring interactions between lncRNAs and candidate genes previously identified via GWAS provides a new functional perspective on the genetic architecture of reproductive aptitude in horses. This approach offers a promising strategy to overcome the inherent limitations of traditional selection, such as low heritability and the scarcity of phenotypic records, by identifying functional markers that could enhance the accuracy of genetic evaluations within equine breeding programs. Recent studies in equine models further underscore this potential, highlighting the dynamic expression of lncRNAs across various reproductive tissues. For instance, Liu et al. [37] identified differential lncRNA expression patterns in the testicular tissue of Mongolian horses at different maturity stages, while Shen et al. [22] revealed a complex whole-transcriptome landscape in the ovarian cortex associated with seasonal reproduction. In this context, the objective of the present study was to predict potential DNA:RNA triplex interactions between lncRNAs and protein-coding genes associated with reproductive efficiency in PRE mares, integrating genomic context and structural accessibility to identify high-viability regulatory candidates capable of modulating the expression of target genes.

### 3.1. Genomic Targets and Candidate LncRNA Selection

Fifteen genes associated with female fertility in PRE horses were selected based on the GWAS published by Laseca et al. [4], namely *HTRA3* (HtrA serine peptidase 3), *SPIRE1* (spire type actin nucleation factor 1), *FOXA3* (forkhead box A3), *APOE* (apolipoprotein E), *ERCC1* (ERCC excision repair 1, endonuclease non-catalytic subunit), *RSPH6A* (radial spoke head 6 homolog A), *KLC3* (kinesin light chain 3), *PDPK1* (3-phosphoinositide dependent protein kinase 1), *MEIOB* (meiosis specific with OB-fold), *PAQR4* (progestin and adipoQ receptor family member 4), *PKD1* (polycystin 1, transient receptor potential channel interacting), *IFT140* (intraflagellar transport 140), *PRSS21* (serine protease 21), and *NME3* (nucleoside diphosphate kinase 3). Most of the genes were located on chromosomes 10 and 13, except for *HTRA3* and *SPIRE1*, which were situated on chromosomes 3 and 8, respectively.

To identify potential cis-regulatory elements, we defined genomic windows extending ±50 kb from the TSS of each candidate gene. This 50 kb criterion was adopted to minimize the false-positive rate, which is typically high in in silico predictions [28], while still capturing most cis-regulatory interactions. Within these windows, lncRNAs were identified for four genes previously linked to mare fertility [4]: *HTRA3*, *ERCC1*, *FOXA3*, and *PRSS21* (Table 1). Both *HTRA3* and *PRSS21* encode enzymes with serine protease activity. Specifically, *HTRA3* plays a role in different stages of ovarian development, driving the differentiation of granulosa cells into luteal cells. In contrast, *PRSS21* (or testisin) is involved in sperm capacitation and has been associated with angiogenesis in the corpus luteum [4,38,39]. Furthermore, the product of *FOXA3* is a transcription factor that interacts with histone proteins in nucleosomes at promoter regions, thereby assisting the remodeling of the chromatin into an active state [40,41]. Finally, *ERCC1* encodes a component of an endonuclease involved in DNA repair mechanisms during DNA replication [42].

Having defined these candidate genes, we investigated potential regulatory lncRNAs located within their genomic windows. A total of eleven lncRNA loci were identified: four in the proximity of *ERCC1*, three each with *HTRA3* and *PRSS21*, and one with *FOXA3* (Table 1). Analysis of the genomic coordinates revealed that three significant GWAS SNPs are physically located within the loci of the candidate lncRNAs. Specifically, AX-103100010 (associated with *HTRA3*) is located within ENSECAG00000053540 (*lnc117842*), AX-103385292 (associated with *ERCC1*) is located within ENSECAG00000057790 (*lnc129946*), and AX-103549135 (associated with *PRSS21*) is located within ENSECAG00000046808 (*lnc140240*). The localization of these fertility-associated variants directly within the lncRNA loci suggests that they may function as causal regulatory variants rather than acting merely as neutral markers in linkage disequilibrium. Genetic polymorphisms mapping to non-coding RNA loci have been previously shown to affect RNA secondary structure and stability, thereby disrupting specific functional motifs required for chromatin interaction and gene regulation [43]. Thus, if these allelic variants disrupt the stability of predicted triplex interactions at the *HTRA3*, *ERCC1*, or *PRSS21* promoter regions, they could plausibly account for the GWAS associations by providing a direct mechanistic link. In this model, the variants impair a cis-acting lncRNA-mediated regulatory switch, ultimately disrupting the precise regulation of genes involved in reproductive fitness [44].

Regarding their annotation status, all eleven identified lncRNA loci are currently classified as “novel genes” in the Ensembl database, a finding consistent with recent whole-transcriptome studies in horses, where up to 85% of the non-coding landscape remains unannotated [22,37].

Consistent with a cis-acting regulatory model, the genomic positioning of the identified lncRNAs relative to their target genes further supports their functional relevance. The distance with the target genes ranged from 1.2 to 49.8 kb, which aligns well with the 10–100 kb proximity criterion applied in recent studies to identify functional lncRNAs in equine testis [37,45], ovaries [21], and placenta [24].

An example of a well-known cis-acting lncRNA is *Chaserr* (CHD2 helper adjacent suppressive regulatory RNA), which is transcribed just upstream of the gene it regulates, *Chd2* (Chromodomain-helicase-DNA-binding protein 2) [46]. However, some cis-regulatory lncRNAs have been shown to regulate genes at greater distances, frequently mediated by the presence of loops and other topological features in the chromatin. For instance, lncRNA *Peril* regulates two genes found at ~1.5 Mb. The most extreme example is *XIST*, a lncRNA involved in the inactivation of the whole X chromosome [44].

### 3.2. Triplex Formation Prediction and Genomic Regulatory Context

We investigated potential interactions between selected fertility-associated genes and nearby lncRNAs using LongTarget. This software predicts all possible DNA:RNA triplexes based on Hoogsteen and reverse Hoogsteen base-pairing and identifies the triplex-forming oligonucleotide with the highest density of overlapping binding sites (TFO1). To assess specificity and distinguish meaningful interactions from background noise, we performed parallel analyses with shuffled lncRNA sequences, shuffled genomic regions, and the promoter of a control gene located on a different chromosome (*MSTN*, myostatin, ECA18). These permutation and control datasets set a reference for distinguishing true interactions from random matches.

The results, summarized in Table 2, show that certain gene-lncRNA pairs generate considerably more overlapping triplexes than their corresponding shuffled or control sequences, indicating that the interaction is sequence-dependent. For instance, the *PRSS21*-*lnc140240* pair exhibited the most robust interaction, characterized by a dense cluster of 34 overlapping triplexes within the promoter region of *PRSS21*. In contrast, randomization of either the lncRNA or the promoter regions largely abolishes the interaction, causing a drastic reduction in the number of triplexes (1 and 2, respectively). Furthermore, it appears to be highly selective for the *PRSS21* locus, as the lncRNA generated only 6 overlapping triplexes when mapped to the promoter of the control gene *MSTN*. This ~4.4-fold enrichment (22/5 = 4.4) over the biological negative control suggests that the high binding density is specific to the *PRSS21* target site rather than a general propensity of the lncRNA to bind genomic promoters.

In contrast, other pairs did not show noticeable differences compared to controls (e.g., *PRSS21*-*lnc86008*) or even exhibited a higher density of predicted triplexes in the randomized sequences (e.g., *ERCC1*-*lnc85946*), suggesting they do not form stable DNA:RNA triplex interactions.

All the analyses were performed using the parameters described in Section 2, with a minimal length of 50 bp (-lg 50) and minimal stability and identity thresholds of 1 (-S 1) and 60% (-i 0.6) to prioritize high-confidence triplexes over low-probability interactions, as recommended by He et al. [33].

To determine the biological potential of these interactions, we analyzed the biophysical properties of the TFO1 sequences. Detailed information is available in Table S1 of the Supplementary Materials. The global mean stability was 1.67, with an average identity score of 65.4%. In this context, LongTarget evaluates the density of possible triplexes at specific positions to determine the binding site with the highest score [33]; therefore, the number of hits can serve as a reliable indicator of the strength of the interaction [47]. Furthermore, the identity score obtained was consistent with previous results showing that certain base mismatches are allowed in Hoogsteen base pairings, providing greater flexibility than the canonical Watson–Crick pairs. This inherent flexibility has been proposed as a mechanism that enables lncRNAs to interact with different genes, or even distinct positions within a gene [19,48].

Meanwhile, the sizes of the interaction sites varied significantly, ranging from 51 to 150 nucleotides, with a mean length of 74.0. Notably, the minimum observed size was substantially larger than the experimental minimum reported by Kunkler et al. [48], who observed that triplexes shorter than 19 nucleotides were generally unstable and failed to form triplexes. Taken together, these data constitute a strong indicator of potential functionality [33].

Additionally, the genomic context of the target genes was analyzed using annotation data from the Ensembl database to determine whether the putative lncRNA binding sites coincide with regulatory regions such as CpG islands, regions rich in CG dinucleotides frequently associated with gene promoters [49]. While the annotation of promoters for the equine genome is less comprehensive than in humans, mice, or cattle, Ensembl provides valuable information on predicted CpG islands, allowing for preliminary insights into potential regulatory interactions.

We identified putative lncRNA-binding sites that overlap with CpG islands for most pairs (Table 3), except for *ERCC1*-*lnc90102*. The pair with the highest number of hits overlapping with CpG islands was *HTRA3*-*lnc82066*, with 24 overlapping hits, followed by *PRSS21*-*lnc140240*, and *HTRA3*-*lnc117842*. Other pairs showed a limited number of predicted TFOs co-located with these regions, particularly *ERCC1*-*lnc85946* and *ERCC1*-*lnc134182*.

The results suggest a potential regulatory role for some of these lncRNAs. This aligns with He et al. [33], who noted that lncRNA binding sites are commonly found within CpG islands. Furthermore, a recent study on equine promoter methylation revealed the importance of these regions for transcriptional regulation in horses [50], where epigenetic mechanisms remain poorly understood.

A proposed mechanism of action involves lncRNAs acting as scaffolds or guides to recruit proteins that modify the methylation status of DNA or histones, such as DNMT3b (DNA-methyltransferase 3 beta) and PRC2 [14]. For instance, the lncRNA *PARTICLE* guides PRC2 to the *MAT2A* promoter, resulting in transcriptional silencing [14,47]. Our results suggest that the identified lncRNAs may similarly act as intermediates in the epigenetic control of equine fertility genes.

However, the enrichment of triplexes at CpG islands does not necessarily imply the recruitment of epigenetic modifiers and they may also regulate transcription through alternative mechanisms. For instance, triplexes can act as molecular anchors that stabilize chromatin loops to facilitate enhancer-promoter communication, as observed with the lncRNA *UMLILO* [44,51]. Alternatively, lncRNAs binding to promoter CpG islands may function via competitive binding, physically blocking the access of transcription factors to the DNA [47,51]. Therefore, while the CpG overlap highlights a regulatory potential, the precise mode of action, whether epigenetic recruitment, spatial reorganization, or competitive blockade binding, requires experimental validation.

### 3.3. Secondary Structure-Based Accessibility of Candidate LncRNAs

The accessibility of the candidate lncRNAs was assessed using RNAplfold, from the ViennaRNA package (v2.7.0) [35]. This software calculates the local base-pairing probabilities (P_unp_) for each nucleotide. Based on this information, it is possible to predict regions that are single-stranded within the secondary structure of RNA molecules based on thermodynamic parameters. This step is critical because the formation of DNA:RNA triplexes via Hoogsteen base-pairing requires the RNA strand to be unpaired to accommodate itself within the major groove of the DNA duplex [14,19].

Single-stranded regions compatible with triplex formation sites predicted by LongTarget were identified for all lncRNAs (Table 4), except for *lnc86008* and *lnc92675*. We found that the total number of overlapping triplexes varied widely, from 35 triplexes within CpG islands for *lnc129946* to 1 for *lnc85946*. Some of these accessible regions, especially in *lnc129946*, *lnc82066*, and *lnc140240*, represent strong candidates for triplex formation, as structural exposure is a requirement for establishing Hoogsteen interactions between the RNA and the DNA double helix. In contrast, the low accessibility of *lnc85946* (1 hit) indicates that the predicted TFO resides in a structurally unavailable region of the RNA, which may limit its ability to form a triplex.

By applying this structural constraint, the false-positive rate is expected to decrease, further supporting the regulatory potential of the top-ranked lncRNAs. This approach is biologically accurate, given that triplex formation must overcome the thermodynamic stability of RNA secondary structures, and the energetic cost associated with the unfolding of RNA regions constituting loops or other structures may pose a barrier [14]. In a previous study, it was proposed that including structural data into the triplex prediction pipeline significantly increased the accuracy of the prediction. The authors compared the results obtained by two different software, Raccess and RNAplfold, concluding that the second one had the best predictive power. Notably, they demonstrated that a threshold of 0.5 of P_unp_ yielded the highest prediction accuracy for lncRNA *MEG3* [19].

### 3.4. High-Confidence Gene-LncRNA Interactions for Equine Fertility Regulation

To identify high-confidence gene-lncRNA interactions, we applied an integrative approach. lncRNAs located within ±50 kb of the TSS of each candidate gene were first selected, consistent with cis-acting regulatory ranges. Predicted DNA:RNA triplexes were evaluated to identify high-confidence binding sites, and their genomic context was analyzed for overlaps with CpG islands. Finally, the structural accessibility of the DNA-binding regions in the lncRNAs was examined to confirm compatibility with triplex formation.

By integrating genomic proximity, triplex enrichment, CpG island overlap, and secondary-structure accessibility, the initial set of candidate interactions was refined, which allowed for the identification of a subset of high-confidence gene-lncRNA regulatory pairs with the strongest potential biological relevance.

We applied a hierarchical filtering strategy, where the primary criterion used for selection was the density of overlapping triplexes in the predicted TFO1. As established by the LongTarget methodology, regions with a higher number of overlapping triplexes are considered to exhibit greater binding potential and specificity, reflecting a stronger likelihood of biologically relevant interactions [33]. This was further supported by the permutation and negative controls, which confirmed that the observed high density was sequence-specific and not the result of random matches. Second, we filtered the enriched candidates based on RNA structural accessibility. The establishment of Hoogsteen base pairs is incompatible with Watson–Crick hydrogen bonds that participate in the formation of loops and other elements. Regions deeply buried within the structure represent an energetic barrier that likely prevents DNA:RNA triplex interactions [19]. However, lncRNAs may operate through alternative mechanisms, including transcriptional interference, enhancer competition, the modulation of local 3D chromatin architecture via chromatin looping, and the direct recruitment of chromatin-modifying complexes via lncRNA-protein interactions [44].

Based on these criteria, three gene-lncRNA pairs emerged as the most robust candidates: *PRSS21*-*lnc140240*, *HTRA3*-*lnc82066*, and *ERCC1*-*lnc129946*. These interactions displayed (1) a high density of overlapping triplexes compared to randomized and control datasets, (2) a high proportion of triplex-forming oligonucleotides located within structurally accessible regions, and (3) substantial overlap with CpG islands in promoter-proximal regions. Together, these features suggest that these lncRNAs may constitute stable triplexes with their target genes.

**Figure 1:**
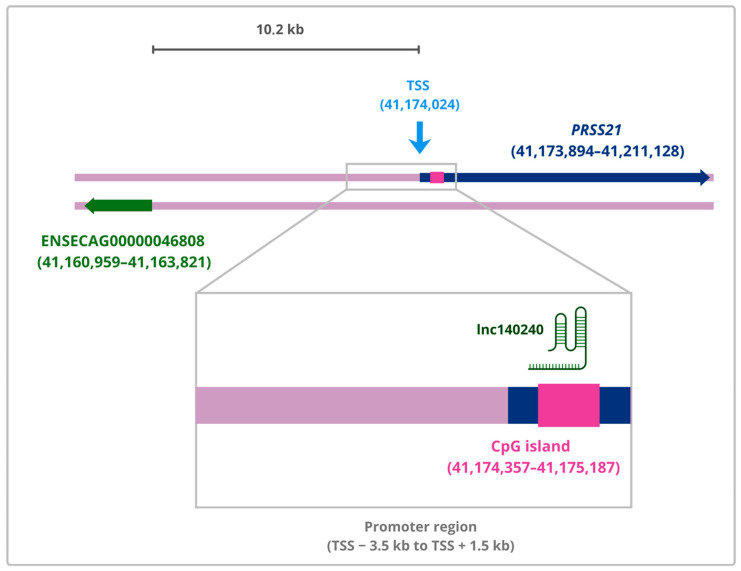
Genomic architecture of the high-confidence *PRSS21-lnc140240* triplex interaction. The target gene is represented by a horizontal dark blue line (coordinates 41,173,894–41,211,128, ECA13), with the Transcription Start Site (TSS), in light blue, identified at coordinate 41,174,024. The *lnc140240* locus is represented as a horizontal dark green line (coordinates 41,160,959–41,163,821), with its transcript depicted as a secondary structure that interacts with a genomic target site located within a CpG island (magenta rectangular box). This regulatory region spans from 41,174,357 to 41,175,187 in the *PRSS21* promoter region.

Among these, *PRSS21*-*lnc140240* represents the strongest candidate for interaction. The dense clustering of triplexes within the *PRSS21* promoter, combined with selective enrichment relative to the *MSTN* control, high RNA accessibility, and overlapping with a CpG island, suggests a targeted and sequence-specific regulatory mechanism (Figure 1). The product of *PRSS21*, testisin, is a serine protease essential for sperm capacitation, which involves the proteolytic cleavage of surface components in the sperm cells. This sequence of events is essential for promoting the interaction with the oocyte and, therefore, the fertilization process in different species, including horses [4,38]. While the specific function of *PRSS21* in the equine ovary remains to be characterized, studies in mouse models have shown that it regulates endothelial integrity and vascularization in reproductive tissues, as its deficiency leads to vascular leakage and failure of the corpus luteum [39]. However, it should be noted that, to our knowledge, there are no specific studies demonstrating *PRSS21* expression or function in the mare ovary or uterus. Consequently, the proposed role of *PRSS21* in equine luteal vascularization is a cross-species hypothesis extrapolated from mice and requires experimental validation.

Additionally, the localization of a SNP directly within the *lnc140240* loci (EN-SECAG00000046808) suggests that this genetic variant may directly modulate the regulatory function of the transcript rather than acting merely as a linked marker. Single nucleotide polymorphisms within lncRNA sequences have been previously shown to alter RNA secondary structure, thereby disrupting specific functional motifs required for chromatin interaction [43]. Furthermore, in the context of DNA:RNA triplex formation, even single base pair mismatches within a TFO can reduce binding affinity or even interfere with the interaction entirely [48,52]. Consequently, if this specific allele disrupted the predicted triplex formation with the *PRSS21* promoter, it would provide a mechanistic explanation for the GWAS signal, linking the genetic variant to the dysregulation of a gene essential for reproductive fitness via a cis-acting lncRNA switch [44].

Previous transcriptional studies in horses have addressed the differential expression of lncRNAs in different tissues associated with the reproductive function, including the testis [45], ovarian cortex [22], granulosa cells [21], oocytes [53], and placenta [24]. In these cases, lncRNAs were assigned biological roles by predicting their target genes based on genomic location. However, no common target genes or lncRNAs have been found between the candidates reported in these datasets and the high-confidence interactions identified in the present study. One of the factors that may be responsible for this discrepancy is the high temporal and spatial specificity of lncRNA expression, which depends greatly on developmental stage and tissue type. Methodological differences may also account for the lack of overlap between the results. These authors relied exclusively on distance to identify target genes for the candidate lncRNAs, with values ranging from 10 to 100 kb. However, this criterion alone does not ensure the identification of biologically relevant interactions. In contrast, our study incorporated the prediction of DNA:RNA triplex formation, together with biophysical constraints inherent to lncRNA secondary structure and the genomic context of target genes, to prioritize interactions with the highest regulatory potential. Finally, the absence of a unified nomenclature for equine lncRNAs further complicates comparisons across studies.

### 3.5. Limitations and Future Directions

While this study identifies high-confidence regulatory lncRNA candidates for equine fertility and supports the hypothesis that lncRNAs may contribute to fertility regulation through DNA:RNA triplex formation, some limitations inherent to the computational design must be considered. First, our results are purely computational predictions and have not been experimentally validated. Definitive confirmation requires in vitro and in vivo assessment of the binding between the gene promoter and lncRNA, as well as functional studies to determine whether these interactions influence gene expression and phenotype. Additionally, the algorithms used for triplex prediction (LongTarget) and structural modeling (RNAplfold) rely on thermodynamic parameters that may contain inherent errors and do not fully account for the chromatin environment, nor subcellular localization or expression levels of the lncRNAs.

A higher precision in genetic selection could be achieved by focusing on variants that directly regulate gene function. Notably, our analysis revealed that the significant GWAS SNPs tagging the *HTRA3* (AX-103100010), *ERCC1* (AX-103385292), and *PRSS21* (AX-103549135) regions are physically located within the loci of some of the identified lncRNAs (*lnc117842*, *lnc129946*, and *lnc140240*). Consequently, experimental validation is required to confirm their regulatory impact. Once this mechanistic link is established, the specific alleles of these lncRNAs that favor stable triplex formation could be incorporated into breeding panels as functional markers, allowing for the selection of mares with optimized epigenetic regulation of key fertility genes, rather than relying solely on linkage disequilibrium with anonymous SNPs.

## 4. Conclusions

This work represents the first computational prediction of cis-lncRNAs in horses that may regulate genes associated with reproductive efficiency via DNA:RNA triplex formation. High-confidence gene-lncRNA interactions mediated by triplex structures were identified as candidate regulatory elements within fertility-associated genomic regions in the PRE horse. By integrating triplex formation scores, structural accessibility, and promoter-specific localization (CpG islands), we prioritized biologically plausible lncRNAs that may contribute to the regulatory architecture underlying reproductive performance.

These findings propose mechanistic hypotheses linking non-coding regulatory elements to equine fertility, a trait characterized by its low heritability and complex genetic architecture. However, the practical implementation of these lncRNAs into genomic selection programs requires further validation, including confirmation of genetic polymorphism within or near these loci, demonstration of association with fertility phenotypes in independent populations, and functional validation of their regulatory activity.

Accordingly, these lncRNAs should be considered candidate regulatory elements that require additional genetic and functional characterization. If experimentally validated, regulatory variants within these loci could be integrated into functionally informed genomic prediction models aimed to improve reproductive efficiency in equine breeding programs.

## Figures and Tables

**Table 1:** Distance between target genes and lncRNA loci located within ±50 kb windows.

Candidate Gene	ECA	lncRNA Gene ID	lncRNA Transcript ID	lncRNA Internal ID	Distance (bp)
*ERCC1*	10	ENSECAG00000057790	ENSECAT00000129946	*lnc129946*	1194
		ENSECAG00000047155	ENSECAT00000085946	*lnc85946*	5844
		ENSECAG00000054326	ENSECAT00000134182	*lnc134182*	10,607
		ENSECAG00000047096	ENSECAT00000090102	*lnc90102*	17,024
*HTRA3*	3	ENSECAG00000053540	ENSECAT00000117842	*lnc117842*	7260
		ENSECAG00000051529	ENSECAT00000092675	*lnc92675*	38,239
		ENSECAG00000050690	ENSECAT00000082066	*lnc82066*	43,648
*PRSS21*	13	ENSECAG00000046808	ENSECAT00000140240	*lnc140240*	10,203
		ENSECAG00000052223	ENSECAT00000086008	*lnc86008*	10,786
		ENSECAG00000051868	ENSECAT00000143931	*lnc143931*	49,817
*FOXA3*	10	ENSECAG00000056523	ENSECAT00000122996	*lnc122996*	39,064

Note: ECA indicates *Equus caballus* chromosome number. Distances represent the absolute genomic distance (in base pairs, bp) between the TSS (transcription start site) of candidate genes and the corresponding lncRNA loci, based on EquCab3.0 reference genome.

**Table 2:** Overlapping triplex counts defining TFO1 for selected gene-lncRNA pairs and corresponding randomized and control sequences.

Gene	lncRNA	Target	Shuffled lncRNA	Shuffled Target	Control
*ERCC1*	*lnc129946*	35	4	2	14
*HTRA3*	*lnc82066*	25	11	2	1
*PRSS21*	*lnc140240*	22	1	2	5
*PRSS21*	*lnc143931*	17	2	1	2
*HTRA3*	*lnc117842*	14	8	1	1
*ERCC1*	*lnc134182*	9	2	1	2
*HTRA3*	*lnc92675*	8	2	1	1
*FOXA3*	*lnc112996*	6	2	2	3
*ERCC1*	*lnc90102*	3	1	1	0
*ERCC1*	*lnc85946*	3	9	1	3
*PRSS21*	*lnc86008*	2	2	1	1

**Table 3:** Number of hits overlapping CpG islands for each gene-lncRNA pair.

Gene-lncRNA Pair	Hits Overlapping CpG Islands
*HTRA3*-*lnc82066*	24
*PRSS21*-*lnc140240*	15
*HTRA3*-*lnc117842*	14
*HTRA3*-*lnc92675*	7
*FOXA3*-*lnc122996*	5
*PRSS21*-*lnc143931*	5
*ERCC1*-*lnc129946*	4
*ERCC1*-*lnc134182*	0
*ERCC1*-*lnc85946*	0
*PRSS21*-*lnc86008*	0
*ERCC1*-*lnc90102*	0

**Table 4:** Number of hits overlapping accessible regions within the lncRNA structure (P_unp_ ≥ 0.5).

lncRNA ID	Hits Overlapping Accessible Regions
*lnc129946*	35
*lnc82066*	25
*lnc140240*	22
*lnc143931*	17
*lnc117842*	14
*lnc134182*	9
*lnc122996*	6
*lnc90102*	2
*lnc85946*	1
*lnc86008*	0
*lnc92675*	0

## Data Availability

The datasets supporting the findings of this study are included in this article and its Supplementary Material. In addition, the genomic data (genes) used are available in the article by Laseca et al. [4].

## Supplementary Materials

The following supporting information can be downloaded at: https://www.mdpi.com/article/10.3390/ani16060898/s1, Table S1: Predicted Class 1 Triplex-Forming lncRNA Interactions with Candidate Reproductive Genes in the Equine Genome.

## Abbreviations

The following abbreviations are used in this manuscript:

lncRNA	Long non-coding RNA
GWAS	Genome-Wide Association Study
PRE	Pura Raza Española
ANCCE	Asociación Nacional de Criadores de Caballos de Pura Raza Española
TSS	Transcription start site
RE	Reproductive efficiency
